# Physical Activity and Ecological Means of Transport—Functional Assessment Methodology

**DOI:** 10.3390/ijerph19159211

**Published:** 2022-07-28

**Authors:** Sylwia Agata Bęczkowska, Iwona Grabarek, Zuzanna Zysk, Katarzyna Gosek-Ferenc

**Affiliations:** 1 Faculty of Transport, Warsaw University of Technology, 00-661 Warszawa, Poland; iwona.grabarek@pw.edu.pl (I.G.); zuzanna.zysk@pw.edu.pl (Z.Z.); 2 Orthopedic Clinic and Rehabilitation, Medical University of Warsaw, 02-091 Warszawa, Poland; kgosek@gmail.com

**Keywords:** physical activity, environmentally friendly means of transport, rehabilitation

## Abstract

Medical developments have led to lower mortality rates but have increased the proportion of people with disabilities or mobility dysfunctions. A higher level of awareness of the general need to perform physical activity, in different spheres of life and at any age, is necessary. A device produced in response to the growing need of supporting active participation in activities of daily life is the Torqway. The aim of this research conducted at the Faculty of Transport, Warsaw University of Technology, was to evaluate the kinematic of users’ upper and lower body muscles motion while riding the tested device. The research was carried out using the MyoMotion system on a stand designed for the purpose of the experiment. Analysis of the results showed a high level of activity in the muscles of the upper limbs, demonstrating that the device can be used to train muscle strength and mass, prevent muscle atrophy, improve the elasticity of periarticular soft tissues and improve the action of the muscle pump (increasing blood flow to the muscles and, with it, the flow of oxygen and nutrients, which promotes the development and subsequent regeneration of muscles). The device can not only serve as a form of recreation but also be used to promote convalescence.

## 1. Introduction

In recent years, there has been a steady upward trend in the development of ecological means of transport [1,2].

Everyday journeys, not only in large conurbations, involve hours spent in traffic jams, often experiencing aggression on the road, as well as being exposed to millions of pollutants emitted into the atmosphere [3,4,5,6].

Harmful substances emitted by road transport are a key factor causing strain on health and danger to life. According to statistics, they cause 385,000 deaths worldwide [7]. Car transport, on the one hand, has many advantages and, on the other hand, poses risks to the environment and people [8]. The search for alternative solutions, providing a range of benefits to both people and the environment, is nowadays the responsibility of designers. Ecotransport is not only a way to reduce pollution, but also a way to spend time, improve one’s physical conditions or participate in the social life of people with different dysfunctions [9,10,11,12].

Electric vehicles are the future of transport [13,14]. In addition to electric cars, personal means of locomotion such as Segway, skateboards, scooters, bicycles or Torqway vehicles are entering the market [15,16]. Currently, there are a number of facilities that stimulate the development of personal electric vehicles thanks to, among other things, project funding from the European Union.

These vehicles ideally complement cars and the public transport system. They are mainly used for short distances, allowing covering them faster than by walking. The developing technology of ecological individual means of locomotion is also conducive to the development of civilisation in society. It causes a number of facilitations and conveniences connected with functioning in everyday life. It also increases the level of activation and presence of people with reduced mobility in various spheres of private and professional life.

## 2. Overview of Individual Environmentally Friendly Means of Transport

Around the world, motor vehicles have long been considered superior to other modes of transport. This is due to the many advantages of the car such as its flexibility, the possibility of door-to-door transport, thermal efficiency. In recent times, however, the advantages of the car over other means of transport, especially in urban environments, have become questionable. It has become increasingly obvious that cars are not the fastest vehicles and that there are more disadvantages than advantages in using them. Rising parking charges have increased the cost of using a car, and the lack of parking spaces has also caused more inconveniences for drivers. In addition, cars are a major source of environmental noise, which has a negative impact on our health. It is associated with, among other things, sleep disorders, irritability, cardiovascular and metabolic diseases, perinatal and pregnancy complications, cognitive impairment, deterioration of mental health and well-being and premature deaths.

Long-term exposure to traffic noise triggers a permanent stress response that results in the release of stress hormones, increased heart rate, increased blood pressure and vasoconstriction, ultimately leading to various chronic diseases [17,18]. In order to minimise the above negative effects, alternative, modern, environmentally friendly means of transport are being sought. These can be divided into electric, human-powered and hybrid vehicles [19,20,21].

This article refers to the most popular and modern alternative vehicles. The first group includes Segway, electric scooters, electric skateboards, while the second group includes bicycles, regular scooters and Torqway vehicles. Individual hybrid vehicles are electric bicycles and Torqway vehicles with additional assistance provided by an electric motor.

The most common individual means of urban transport are scooters and bicycles. In Poland, as in other countries, the popularity of electric scooters has grown rapidly. They were first introduced to the streets of Warsaw and Wrocław in 2018 by Lime [22]. Already in 2019, nine companies offering to rent them were operating in the country, and more than 11,000 such unicycles appeared on the streets. Their number continues to grow. Lime claims that just one year of operation, its vehicles had travelled almost 7 million kilometres travelled [23]. In turn, Bolt, another scooter rental system operator, intends to offer its services in more than 100 cities in Europe, making available over 130,000 electric scooters and bicycles [24]. Thanks to its wide tyres, an electric scooter can move smoothly on uneven surfaces such as pavement slabs. It is a more comfortable and safer means of transport than a traditional scooter. Other popular means of transport in cities are electric bicycles and scooters [25]. Estimates of recent changes in urban transport mode preferences in the USA show that e-scooters could also replace up to 1% of taxi trips in central urban areas [26].

These vehicles are also slowly making their way into Polish cities—they can be hired in Warsaw, for example. In the case of bicycles, a small number of them are available within the urban bicycle network. Regular bicycles still have their adherents, e.g., in the Netherlands and other cities, and are an unbeatable means of travel. In addition, they provide the possibility of performing physical activity and therefore have a beneficial effect on users’ health. However, the performance of a bicycle depends on the physical fitness of the cyclist and his/her willingness to expend the high energy needed to reach the destination [27]. Scooters, on the other hand, can be rented similarly to electric scooters, though their renting has been limited so far. At the beginning of the season in 2021, there were about 800 units in 11 cities, with about a third of this number in Warsaw. At the moment, however, the scooter market is not growing as fast as that of electric scooters. There is also a lack of the necessary infrastructure, i.e., places where to charge the scooters and locations designated for their parking [28]. According to forecasts [28], the global personal mobility devices market is expected to grow at least until 2024 with a compound annual growth rate of 7.0%. This figure shows unprecedented potential. North America is currently the leading market, while Europe is in second place with approximately a 35% share [29].

Less popular today is the Segway, originally used in shopping centres, warehouses and airports. Segway is a self-balancing vehicle with a maximum speed of about 20 km/h. The first commercially available model was the Segway HT i167; later, the name of the manufacturing company became widely used to designate products with similar characteristics, even if they were made by other companies. The Segway was introduced in December 2001. From March 2002 to February 2003, only about 6000 units were sold, while the factory was designed to produce up to 40,000 Segway per year [30]. For many years, the Segway that was synonymous with fast and eco-friendly travel in urban traffic [31]. Now, many Segway models are available, although this vehicle is not as popular as scooters.

The countries with the fastest growth in private e-scooter use in 2019 are Spain (498% annual growth), France (132%), Germany, Italy (286%), Poland, Austria, the Netherlands, Belgium and Switzerland [32]. A total of 55% of all electric scooters are located in Spain and France alone, where more than every second scooter is distributed. The provision of electric scooters was implemented in most European countries since 2019. Germany is currently in third place. The main European cities where scooter sharing is implemented are Madrid, Paris, Barcelona, Berlin, Milan, Rome and Nice.

## 3. Research Object

**Figure 1:**
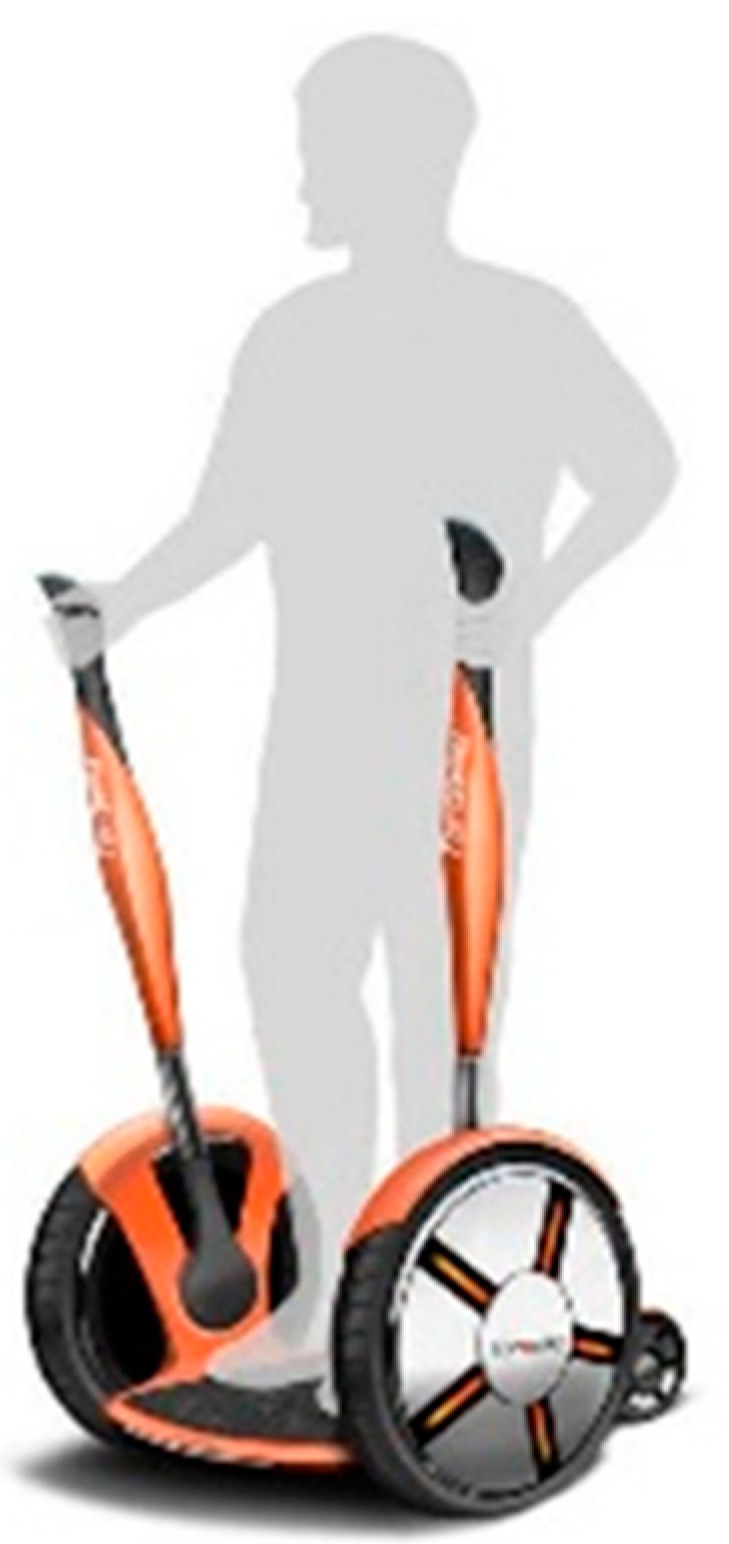
Torqway vehicle. Source: based on https://torqway.com/pl (accessed on 20 May 2022).

A slightly different means of transport is the above-mentioned Torqway, an invention patented in Poland in 2011. It was created first of all for sport and recreation purposes. What distinguishes it from scooters, Segway or electric scooters is the fact that its use involves the muscles of the upper and lower limbs and of the torso. It is a manually propelled vehicle that enables movement in a standing position, similarly to Segway. Moving the lever forwards and backwards while riding generates a unidirectional rotational movement of the wheel axle, causing the vehicle to move forward. The device can be used to ride in the city, in parks, on a bike path or in a shopping centre. It allows driving at speeds of up to 12 km/h. Activity on the Torqway combines elements of Nordic walking and work on an orbiter-type device. As the device is easy to operate, it can be used by children, adults and seniors. The wide range of potential users significantly distinguishes the Torqway from other individual means of transport (Figure 1). A hybrid version is now also available, which can be used by physically weak people.

Propulsion of the vehicle mainly involves movement of the shoulder and elbow joints. The direction of travel is controlled by foot activity. To make a right turn, the pedal must be pressed with the right heel. The outward movement of the lever is used for braking. The length and frequency of the lever movement depend on the user’s will. The resistance of the lever is moderate

As the Torqway is not as popular as the other mentioned means of individual transport, research was undertaken to assess its level of functionality. This vehicle was the subject of research conducted by the Faculty of Transport at the Warsaw University of Technology. The study group consisted of 11 women aged over 50 years.

Taking into account its design and the way it is set in motion, the hypothesis was formulated that the use of this vehicle for recreational purposes may have beneficial effects on the musculoskeletal system, which means that to some extent, the vehicle may be used in rehabilitation exercises. It was also hypothesised that parameters such as muscle tone and range of motion of body segments of a person riding the Torqway could be used to assess its level of functionality.

The experimental research was carried out using the surface electromyography method, which enables the analysis of the activity of muscles involved in driving a vehicle, and the Myo Motion system, which enables the analysis of body kinematics while driving. The research was conducted on a test stand designed for the purpose of the experiment.

The article presents the results of body kinematics obtained using the Myo Motion system. These results are part of an overall assessment of the level of functionality of the Torqway vehicle.

## 4. Materials and Methods

Experimental research on the physical activity of users using the Torqway vehicle (manufacturer Torqway, Toruń, Poland) was carried out on behalf of the TORQWAY company, implementing the construction of the hybrid stand-up mobility vehicle “Torqway” within the framework of the European Union research and innovation programme called Horizon 2020, project number: 778154 (SMEI H2020).

### 4.1. Aim and Scope of the Research

**Figure 2:**
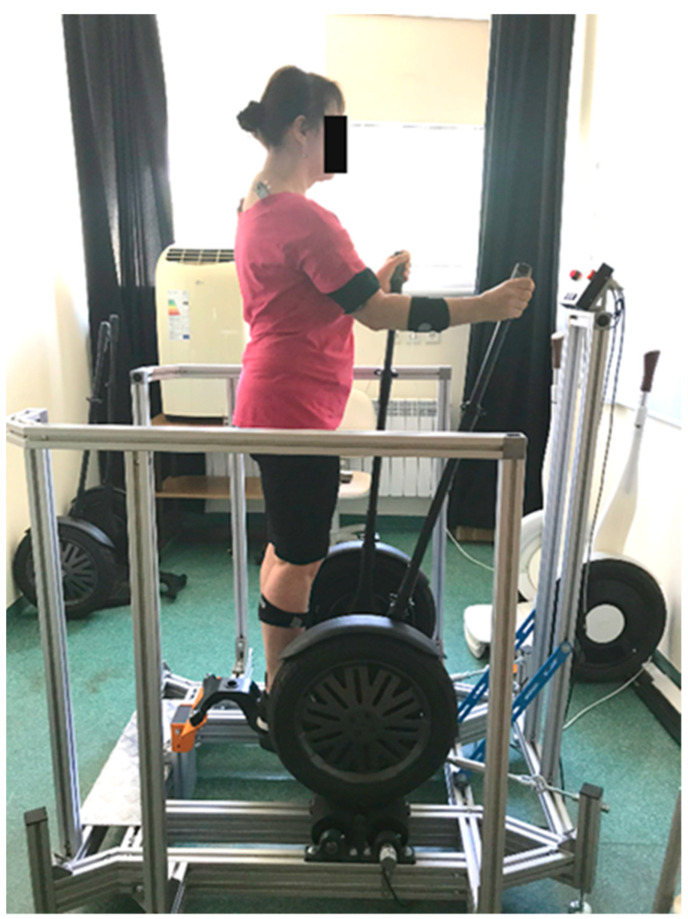
Test stand.

The aim of the research was to assess the activity of the muscles of the upper and lower body. The experimental research was carried out on a specially constructed test stand (Figure 2). The research group consisted of 11 women aged 50+ years. Assumptions regarding age resulted from the conditions of the project. The characteristics of the research group are presented in Table 1.

The mean age was 54.36 years. Due to the small size of the research group, homogeneity was maintained in the selection of participants from the point of view of fitness and physical build.

The participants performed movements with their upper limbs by driving a vehicle immobilised on a roller stand using levers (Figure 2).

The design of the stand allowed the wheels to be driven at different speeds, shown on a display. By ensuring constant driving conditions, it was possible to accurately determine and evaluate the ranges of body motion. The driving conditions corresponded to the “normal” operation of the Torqway vehicle in real conditions. Fifteen anatomical angles of the body elements (joints) were taken into account in the tests in order to determine the range of their work.

List of body parts tested:

- Lumbar Flexion
- Lumbar Lateral
- Lumbar Axial
- Thoracic Flexion
- Thoracic Lateral
- Thoracic Axial
- Elbow Flexion
- Shoulder Total Flexion
- Shoulder Flexion
- Shoulder Abduction
- Shoulder Rotation out
- Hip Flexion
- Hip Abduction
- Hip Rotation out
- Knee Flexion

### 4.2. Description of the Myo Motion System Used for Testing

Noraxon’s MyoMotion ( manufacturer Noraxon, Scottsdale, AZ, USA) system was used in the study [33]. This system is used to assess the movement of body segments in 3D using a set of inertial sensors, each containing an accelerometer, a gyroscope and an earth magnetic field sensor. In addition, it is equipped with sensor mounting accessories and software that enables both data recording and comprehensive data analysis. The system used for the research has a number of advantages useful for functional evaluation studies of various mobile means of transport; namely, it is a completely wireless motion registration system that analyses in 3D changes in angles between segments, sensor orientation and linear acceleration. It is a system used outdoor or indoor. The accuracy of registration of angular changes is +/−1° in static conditions, +/−2° in dynamic conditions, with sampling frequency of 100 or 200 Hz (depending on the configuration). It has an extensive software with implemented models of all main joints, thanks to which it is possible to assess their range of motion or analyse the repeatability of a movement. In the system, each sensor can be assigned to any body segment, and the visualisation of the measurement takes place in real time using an avatar (skeleton).

### 4.3. Test Procedure

In order to examine the angular values at individual joints of the upper and lower limbs, ranges of motion were measured for 9 movements (including flexion, abduction, external rotation) at 4 joints (shoulder, elbow, hip, knee) for both the right and the left side of the body. The determination of the anatomical angles was performed according to the principles of the medical neutral/zero method. It was assumed according to [34,35] that in a human standing upright, all joints are in zero position, even if there is a deviation from the geometric zero angle. For example, the geometric angle at the ankle joint is 90°, and the anatomical angle is 0°. Left rotations and lateral flexions are always negative, right rotations are always positive.

**Figure 3:**
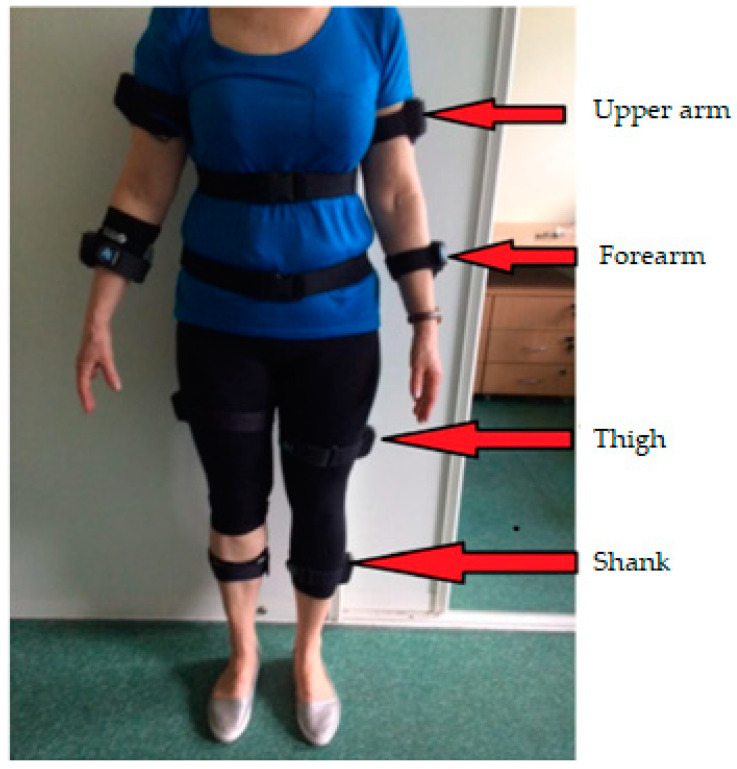
Location of the sensors on the test person.

**Figure 4:**
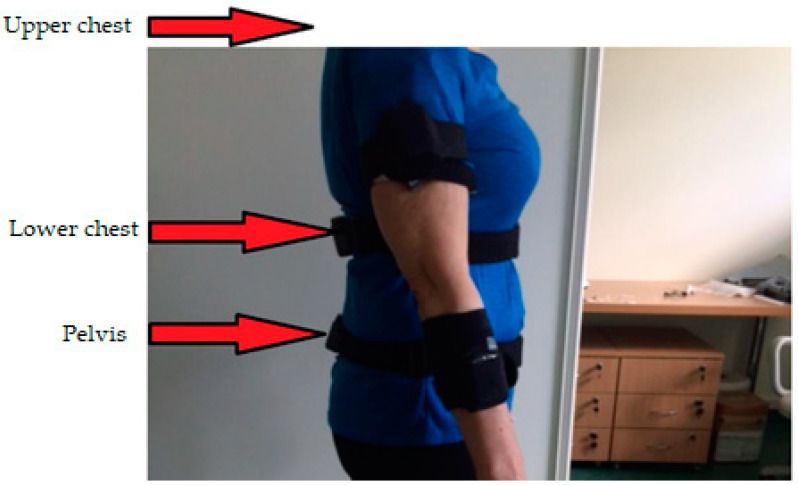
Sensor placement on the test person—side view.

Each sensor used was labelled with the X, Y and Z axis. All sensors were placed on the body segments in such a way that, when standing, the X-axis vector on the sensor label was directed vertically along the axis of gravitational force. It was important to establish a location where the muscle belly in a given segment would not move the sensor relative to the underlying bones. In order to obtain the best possible results, the principles for sensor placement described in [36] were followed. The sensor placement used, along with its description, is shown in Figure 3 and Figure 4, with frontal and sagittal plane views of the subject, respectively.

Changes in spinal angles were measured using 11 motion sensors. Selected properties of the sensors are presented in Table 2.

All sensors were attached to the body via special straps. The sensors wirelessly transmitted information about angular changes in relation to each other to a computer with dedicated software.

Once the sensors were placed in the appropriate places, they were calibrated (in other words, landmarks were digitised). During calibration, the subject stood still in a pre-determined position (upright, feet hip-width apart, hands along the body at shoulder width). The digitisation of the landmarks allows the myoMOTION model to be scaled and positioned to fit the subject and eliminates the assumptions used in standard calibration.

After calibration, the test subject entered the Torqway vehicle, took a starting position and, at a preset cue, began the test. The measurement lasted 3 min (180 s). During the measurement, the subject performed motor activities similar to those performed during natural Torqway driving.

## 5. Results

**Figure 5:**
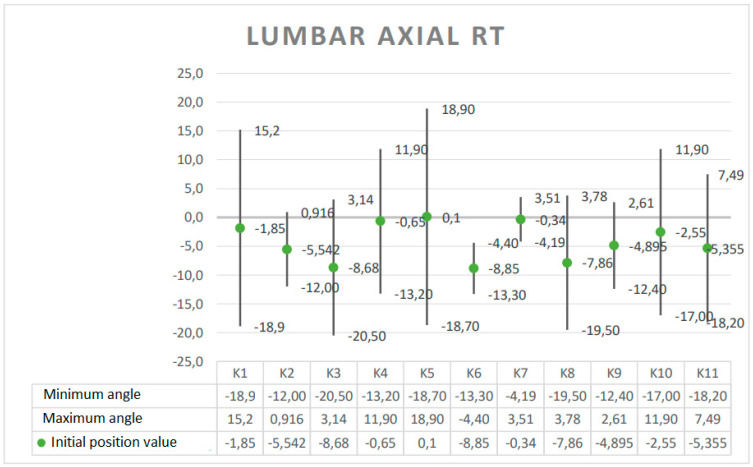
Angle ranges for lumbar rotation.

**Figure 6:**
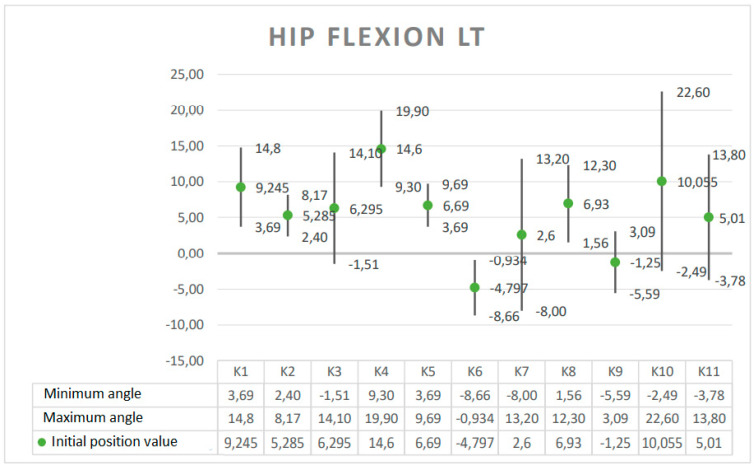
Angle ranges for left hip flexion.

**Figure 7:**
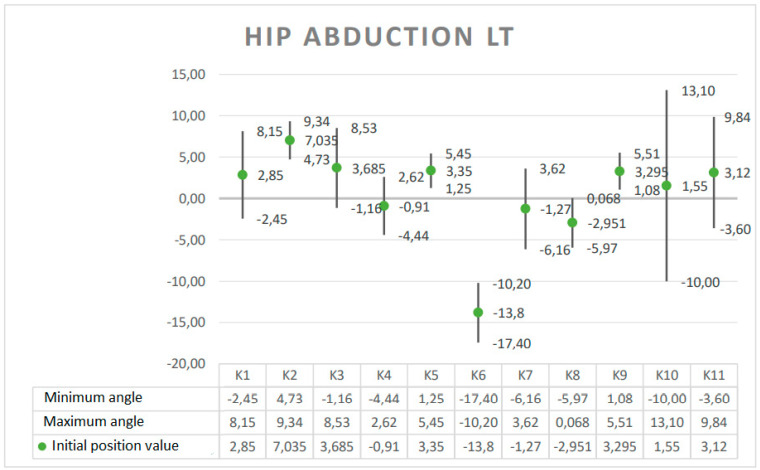
Angle ranges for left hip joint extension.

**Figure 8:**
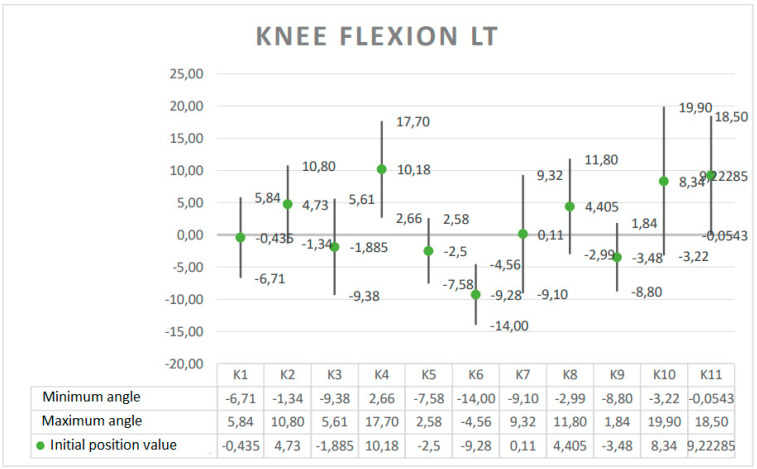
Angle ranges for left knee joint flexion.

**Figure 9:**
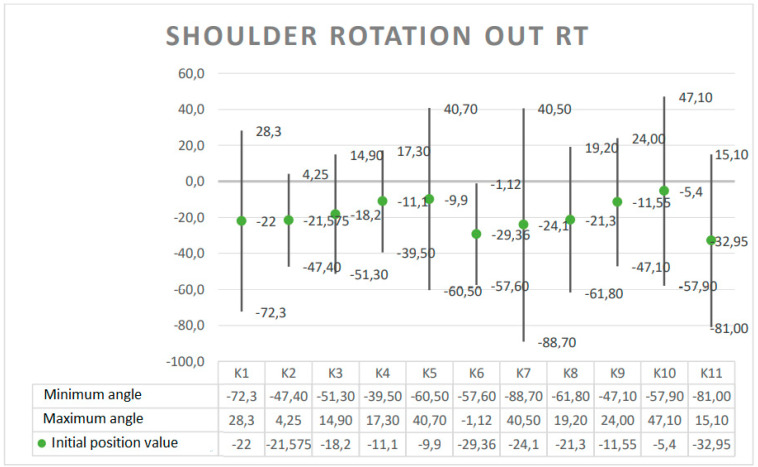
Angle ranges for external rotation of the right shoulder joint.

The results are presented in the form of graphs, which include the minimum and maximum angular value reached at a given joint during the Torqway test and the range of motion (ROM). These data re helpful in planning the use of the device in the rehabilitation process, especially after surgery on individual joints. The results obtained for selected anatomical angles of body elements are presented in Figure 5, Figure 6, Figure 7, Figure 8 and Figure 9.

Table 3 shows the normal ranges of motion in the spine (for the cervical, thoracic and lumbar segments) and in the hips.

The measured values for all study participants are shown in Table 4.

## 6. Discussion

The research presented here involved analysing the kinematics of body movement of a person simulating the movement on a Torqway vehicle on a stationary stand. The measurements were carried out using inertial measurement sensors (IMSs) from Noraxon’s MyoMotion system and were aimed at assessing the level of functionality of the prototype Torqway vehicle. It is worth noting that this type of system can be used to assess the kinematics of movement, allowing the development of solutions that can be used to support the rehabilitation and training evaluation of athletes. The use of MyoMotion to analyse the movement of athletes, e.g., in alpine skiing, has been reported [38]. However, the aim of the aforementioned studies was to evaluate the correct placement of the sensors in order to obtain the best possible feedback signal. Research using the same technique was also performed at the Academy of Physical Education in Poznań [39], and its aim was to develop an appropriate training programme based on the assessment of movement kinematics in athletes. There are also many papers in the medical and biomechanics literature describing the use of the MyoMotion system to assess lower or upper limb movement kinematics after medical procedures, e.g., endoplasty [40,41].

Among the most commonly used systems for the analysis of movement kinematics, we can distinguish optoelectronic tracking systems–multi-camera systems measuring the trajectories of markers placed on the patient’s body (example systems: Vicon, BTS, Optitrack), video systems—markerless systems, recording images in visible light, using image analysis methods (example system: Simi), acoustic systems—using time-of-arrival measurements or phase shifts of the generated ultrasonic waves (example system: Zebris), electronic goniometers—measurement of angles in a joint in a selected plane.

While selecting the system for measurements, it is important to assess the accuracy of inertial sensors measurements during various tasks of everyday life, as formulated by [42]. Their low cost and ease of use make them an attractive option for motion analysis tasks. Moreover, different studies using the MyoMotion system for level walking [43,44,45,46,47,48], long-term ergonomic tasks [49], stair climbing [43,45] and inclined walking [42], showed a good agreement in angle waveform in the sagittal plane. In the non-sagittal planes, the angle waveforms did not agree as well. Measurements of the ranges of movement of body parts on the Torqway vehicle were performed in the sagittal plane, so the choice of the measurement system can be considered correct given, these results.

The stationary tests were designed by minimising the risk of injury during real-world driving, given the insufficient time for training classes prior to the tests. An additional requirement formulated by the principal was the nature of the research group, namely, women aged 50+ years. In formulating the research hypothesis, it was assumed that one of the parameters that can indicate good vehicle functionality is the range of movement of the limbs and of the human body during driving. The greatest differences in anatomical angles could be seen in lumbar rotation, hip flexion and extension, knee flexion and right shoulder joint extension. The most standardised movements were elbow flexion and external rotation of the hip joint. However, all ranges were within the limits accepted as normal. In addition, the design of the vehicle allows each person to adapt the range of motion to their own abilities and preferences, which is why the results of different subjects differ slightly. Analysis of the results showed a change in position in all joints included in the study. The joints of the rim and upper limbs moved more than the joints of the lower limbs and trunk, but movement occurred at every level. As a result, Torqway can be used to improve overall body mobility and overall fitness. The highest ROM values were obtained for the following movements: elbow joint flexion, shoulder joint abduction, shoulder joint flexion [50,51,52,53,54,55,56,57]. The lowest ROM values were obtained for lower limb and spine movements. The minimum and maximum joint angles achieved by a test subject vary from person to person. This means that the body position while riding the Torqway is individually adapted to the capabilities of the subject (due to limitations, comfort, compensations). Given the possibility of using the Torqway vehicle as a rehabilitation device, immobilised on the stand, the possibility of individual adaptation does not exclude any patient from training on this device. The above results entitle us to conclude that our hypothesis about the possibility of using the analysis of limb and human body movement kinematics to assess the level of functionality of the Torqway vehicle is true. A comprehensive assessment of the level of functionality still requires the measurement of muscle tension by surface electromyography and the verification of the truth of the hypothesis. This issue requires a separate discussion in a subsequent article.

It is worth noting that no work was found in the literature on the evaluation of the movement kinematics of users of individual, mobile, environmentally friendly means of transport. The research on the Torqway device was first carried out at the Department of Transport and on a prototype device. The application of the techniques described in this study to test the suitability of individual vehicles from the point of view of user fit and the potential benefits that their use can bring represents a new approach in research that will undoubtedly be pursued.

### Limitations

The sensors used are exposed to soft tissue movements that cause their wobbling and affected the internal IMU. This might lead to the overestimation of the joint angles in the non-sagittal planes. Another drawback of the IMU system is the method of calibration: firstly, it is dependent on the subject to achieve the calibration posture and, secondly, coordinate systems are defined on the segment orientation only. Thereby, the IMU system disregards possible physiological reasons for deviations, e.g., varus or valgus positions of the joints. Additionally, the sensors might be influenced by magnetic distortion, which will lead to an incorrect offset rotation. Both limitations strongly influence the joint angle calculation. This subject was previously discussed [42,43].

## 7. Conclusions

The research presented here was concerned with analysing the level of functionality of a new personalised vehicle, the Torqway. Functionality, as formulated in this case, is a function of parameters defining the range of movement of individual body parts when using the vehicle and the tension of the muscles involved in this movement. Research using the MyoMotion system showed that the ranges of all movements were within the limits assumed to be normal. The participants in the experiment—women aged 50+ years—had no problem realising the reciprocating movements with their upper limbs that generate the vehicle’s movement. The design of the vehicle allows each person to adapt the range of movement to their own abilities and preferences, so the results differ slightly between the subjects.

According to the female participants, the vehicle is easy to use. It can therefore be concluded that the assessment of the functional level from the point of view of the ranges of movement of individual body parts is satisfactory. A comprehensive assessment, according to the hypothesis, still requires muscle tone testing to form an opinion on the use of the Torqway in rehabilitation programs. The results of these studies will be presented in a subsequent article.

Finally, it is worth emphasising that the Torqway not only has the potential to promote the performance of physical activity by people of all ages and thus their health, but also is environmentally and surroundings-friendly, as it does not generate any pollutants into the atmosphere. Therefore, it can be freely ridden both in cities and in fume-free green areas.

## Figures and Tables

**Table 1:** Characteristics of the study group—pilot studies.

Respondent	Age	Height (cm)	Weight (kg)	BMI
k1	54	165	72	26.45
k2	54	164	79	29.37
k3	52	153	62	26.49
k4	59	162	69	26.29
k5	62	165	63	23.14
k6	50	167	90	32.27
k7	57	166	69	25
k8	50	170	57	19.7
k9	52	164	57	21.2
k10	58	161,5	62	23.92
k11	50	170	72	24.91

**Table 2:** Characteristics of the sensors.

	Characteristics
Dimensions	37.6 mm (L) × 52 mm (W) × 18.1 mm (H)
Weight	Less than 34 g
Output & Transmission Frequency	Up to 2.5 mW
	DSSS 2415–2472 MHz on (up to) 8 selectable radio channels
	Utilizing up to 4 different radio frequencies on each channel
	Signal latency of 140 ms during data collection
	sensor transmission range: 30 m
X, Y, Z acceleration sampled at:	Low g accelerometer: 800 Hz
	High g accelerometer: 400 Hz
X, Y, Z angular velocity sampled at:	Low speed gyro: 800 Hz
	High speed gyro: 400 Hz
X, Y, Z magnetic field sampled:	50 Hz
Accuracy	+/−1 degree in vertical plane, +/−2 degrees in horizontal plane
Battery life	8 h, 3 h charge time
Sample rates	100 Hz and 200 Hz
Other	Fully wireless Inertial Measurement Sensor
	2.4 GHz unlicensed radio

**Table 3:** Normal ranges of motion in the spine and hips. Source: based on [37].

	Cervical (°)	Thoracic (°)	Lumbar (°)	Hips (°) (Excluding ab and Adduction)
Flexion	0–60	0–50	0–60	0–110
Extension	0–75	0–45	0–25	0–30
Lateral Flexion	0–45	0–40	0–25	n/a
Rotation	0–80	0–30	0–18	Internal = 0–40 External = 0–50

**Table 4:** Values of measured ranges of movement analysed for all subjects.

	Range of Motion										
	K1	K2	K3	K4	K5	K6	K7	K8	K9	K10	K11
Lumbar Flexion	8.78	11.51	13.09	13.53	10.16	6.79	10.80	8.29	11.14	14.26	20.32
Lumbar Lateral RT	6.09	5.26	7.14	7.19	8.28	5.83	5.42	5.48	4.51	13.50	8.31
Lumbar Axial RT	34.10	12.92	23.64	25.10	37.60	8.90	7.70	23.28	15.01	28.90	25.69
Thoracic Flexion	11.77	11.85	22.72	15.55	16.62	9.36	29.10	14.62	30.70	37.90	33.09
Thoracic Lateral RT	11.66	8.08	17.07	12.16	11.48	9.81	24.07	9.69	16.27	16.16	16.44
Thoracic Axial RT	27.37	11.22	25.59	15.48	14.69	12.34	51.50	29.12	36.19	43.30	16.10
Elbow Flexion LT	120.90	90.20	110.10	117.70	113.36	68.10	105.85	107.35	99.80	115.45	109.38
Elbow Flexion RT	115.30	91.21	108.90	69.80	123.10	59.80	128.50	103.52	79.90	109.50	130.17
Shoulder Total Flexion LT	42.70	39.28	54.11	41.07	47.56	32.50	55.15	39.70	42.80	68.19	63.09
Shoulder Total Flexion RT	51.03	40.42	44.80	41.89	55.61	34.54	52.89	41.26	45.97	57.02	66.42
Shoulder Flexion LT	89.20	60.00	62.83	47.30	58.02	39.66	89.10	69.70	79.30	84.90	86.10
Shoulder Flexion RT	85.90	59.00	53.55	56.50	80.50	42.48	101.90	60.40	90.60	90.60	83.20
Shoulder Abduction LT	58.30	21.23	54.00	52.80	42.10	26.66	66.10	35.55	35.83	53.90	40.30
Shoulder Abduction RT	47.40	22.79	44.03	24.06	66.20	20.35	80.40	33.90	40.00	56.20	51.96
Shoulder Rotation out LT	71.41	56.98	75.30	137.10	50.20	47.79	113.80	61.45	90.60	106.20	77.71
Shoulder Rotation out RT	100.60	51.65	66.20	56.80	101.20	56.48	129.20	81.00	71.10	105.00	96.10
Hip Flexion LT	11.11	5.77	15.61	10.60	6.00	7.73	21.20	10.74	8.68	25.09	17.58
Hip Flexion RT	12.46	8.76	10.79	11.27	8.77	8.48	28.67	12.40	9.74	30.96	14.16
Hip Abduction LT	10.60	4.61	9.69	7.06	4.20	7.20	9.78	6.04	4.43	23.10	13.44
Hip Abduction RT	10.08	5.40	11.51	4.81	4.21	3.49	10.86	7.24	10.12	18.58	11.65
Hip Rotation out LT	74.80	73.60	73.30	78.60	72.00	56.50	66.70	83.00	78.60	85.70	81.50
Hip Rotation out RT	82.30	48.00	111.00	98.00	62.50	81.57	99.20	44.00	50.40	79.70	65.40
Knee Flexion LT	12.55	12.14	14.99	15.04	11.84	9.44	18.42	14.79	10.64	23.12	18.55
Knee Flexion RT	13.18	10.50	14.93	14.16	10.31	7.74	24.51	14.65	16.48	20.47	17.92

## Data Availability

Data are available upon request.

## References

[1] Conserve Energy Future What Is Green Transportation?. https://www.conserve-energy-future.com/modes-and-benefits-of-green-transportation.php.

[2] E-Scooter Findings Report 2018 PBOT (Portland Bureau of Transportation). https://www.portlandoregon.gov/transportation/article/709719.

[3] Siramy A. (2018). How Choice Makes City Transportation Smart and Sustainable. https://www.smartcitiesdive.com/news/how-choice-makes-city-transportation-smart-and-sustainable/515246/.

[4] Quaium R. (2012). Sustainable Urban Transportation Systems, United Nations Economic and Social Commission for Asia and the Pacific ESCAP and CITYNET. https://www.uncclearn.org/sites/default/files/inventory/unescap20_0.pdf.

[5] Edaño D. (2014). Urban Transport Problems. Engineering. https://www.slideshare.net/paojean2000/urban-transport-problems.

[6] https://www.epa.gov/transportation-air-pollution-and-climate-change/history-reducing-air-pollution-transportation.

[7] International Council on Clean Transportation (2019). A Global Snapshot of the Air Pollution-Related Health Impacts of Transportation Sector Emissions in 2010 and 2015.

[8] Khardi S., Bernoud-Hubac N. (2022). Editorial for the Special Issue “Impacts of Transport Systems on Air Pollution and Human Health”. Atmosphere.

[9] Mehlig D., Woodward H., Oxley T., Holland M., ApSimon H. (2021). Electrification of Road Transport and the Impacts on Air Quality and Health in the UK. Atmosphere.

[10] Bęczkowska S.A., Zysk Z. (2021). Safety of People with Special Needs in Public Transport. Sustainability.

[11] Implementation of the World Programme of Action Concerning Disabled Persons [1982] UNGA 68; A/RES/37/53. 3 December 1982. http://www.worldlii.org/int/other/UNGA/1982/57.pdf.

[12] World Bank, World Health Organization (2011). The World Report on Disability. http://www.who.int/disabilities/world_report/2011/en.

[13] Sanguesa J.A., Torres-Sanz V., Garrido P., Martinez F.J., Marquez-Barja J.M. (2021). A Review on Electric Vehicles: Technologies and Challenges. Smart Cities.

[14] International Energy Agency (2021). Trends and Developments in Electric Vehicle Markets. Global EV Outlook 2021.

[15] Torqway. https://torqway.com/en.

[16] Segway. https://pl-pl.segway.com/.

[17] Altinsoy M.E. (2022). The Evaluation of Conventional, Electric and Hybrid Electric Passenger Car Pass-By Noise Annoyance Using Psychoacoustical Properties. Appl. Sci..

[18] Neufville R., Abdalla H., Abbas A. (2022). Potential of Connected Fully Autonomous Vehicles in Reducing Congestion and Associated Carbon Emissions. Sustainability.

[19] Boglietti S., Barabino B., Maternini G. (2021). Survey on e-Powered Micro Personal Mobility Vehicles: Exploring Current Issues towards Future Developments. Sustainability.

[20] Al Mamun A., Zainol N.R., Hayat N. (2020). Electric Scooter-An Alternative Mode of Transportation for Malaysian Youth. https://www.researchgate.net/publication/346686997_Electric_Scooter_-_An_Alternative_Mode_of_Transportation_for_Malaysian_Youth.

[21] Oxley J., Logan D.B., Coxon S., Koppel S. (2022). Understanding Current and Future Transport Needs of Older Australian Drivers to Guide Development of Sustainable and Smart Initiatives to Support Safe Mobility of Older Adults. Sustainability.

[22] Kmag. https://kmag.pl/article/lime-w-polsce.

[23] Lime. https://www.li.me/.

[24] Electro Mobility. https://mycompanypolska.pl/artykul/raport-elektromobilnosc-hulajnoga-do-celu-czyli-wygodny-transport-w-miastach/8015.

[25] Bolt. https://www.bolt.eu/.

[26] Van den Steen N., de Geus B., Cappelle J., Vanhaverbeke L. (2022). Cycling Infrastructure for All EPACs Included?. World Electr. Veh. J..

[27] Reck D.J., Martin H., Axhausen K.W. (2022). Mode choice, substitution patterns and environmental impacts of shared and personal micro-mobility. Transp. Res. Part D Transp. Environ..

[28] Rose G. (2012). E-bikes and urban transportation: Emerging issues and unresolved questions. Transportation.

[29] ReportBuyer Personal Mobility Devices Market—Global Industry Analysis, Size, Share, Growth, Trends, and Forecast, 2019–2027. https://www.reportbuyer.com/product/4142781/personal-mobility-devices-market-global-industry-analysis-size-share-growth-trends-and-forecast-2019-2027.html?utm_source=PRN.

[30] Howe E., Bock B. (2018). InnoZ-Innovation Centre for Mobility and Societal Change (InnoZ). Global Scootersharing Market Report 2018.

[31] Kim B.W., Park B.S. (2016). Robust Control for the Segway with Unknown Control Coefficient and Model Uncertainties. Sensors.

[32] Jamerson F.E., Benjamin E. (2012). Worldwide Electric Powered Two Wheel Market. World Electr. Veh. J..

[33] Zagorskas J., Burinskienė M. (2020). Challenges Caused by Increased Use of E-Powered Personal Mobility Vehicles in European Cities. Sustainability.

[34] Noraxon. https://www.noraxon.com/our-products/myomotion/.

[35] Triantafyllou A., Papagiannis G., Stasi S., Bakalidou D., Kyriakidou M., Papathanasiou G., Papadopoulos E.C., Papagelopoulos P.J., Koulouvaris P. (2022). Application of Wearable Sensors Technology for Lumbar Spine Kinematic Measurements during Daily Activities following Microdiscectomy Due to Severe Sciatica. Biology.

[36] Che-Nan H., Rambely A.S. (2022). Kinematic Analysis of Daily Activity of Touching Lateral Shoulder for Normal Subjects. Appl. Sci..

[37] Ferguson B. (2014). ACSM’s Guidelines for Exercise Testing and Prescription 9th Ed. 2014. J. Can. Chiropr. Assoc..

[38] Pasanen T.P., Tyrväinen L., Korpela K.M. (2014). The Relationship between Perceived Health and Physical Activity Indoors, Outdoors in Built Environments, and Outdoors in Nature. Appl. Psychol. Health Well Being.

[39] Yu G., Jang Y.J., Kim J., Kim J.H., Kim H.Y., Kim K., Panday S.B. (2016). Potential of IMU Sensors in Performance Analysis of Professional Alpine Skiers. Sensors.

[40] Bańkosz Z., Winiarski S. (2017). Kinematics of table tennis racket. Differences between topspin shots. J. Sports Med. Phys. Fit..

[41] Alrawashdeh W., Siebers H.L., Reim J., Rath B., Tingart M., Eschweiler J. (2021). Gait symmetry—A valid parameter for pre and post planning for total knee arthroplasty. J. Musculoskelet. Neuronal Interact..

[42] Di Paolo S., Lopomo N.F., Della Villa F., Paolini G., Figari G., Bragonzoni L., Grassi A., Zaffagnini S. (2021). Rehabilitation and Return to Sport Assessment after Anterior Cruciate Ligament Injury: Quantifying Joint Kinematics during Complex High-Speed Tasks through Wearable Sensors. Sensors.

[43] Mundt M., Thomsen W., David S., Dupré T., Bamer F., Potthast W., Markert B. (2019). Assessment of the measurement accuracy of inertial sensors during different tasks of daily living. J. Biomech..

[44] Mundt M., Wisser A., David S., Dupré T., Quack V., Bamer F., Tingart M., Potthast W., Markert B. The influence of motion tasks on the accuracy of kinematic motion patterns of an imu-based measurement system. Proceedings of the 35th Conference of the International Society of Biomechanics in Sports.

[45] Nüesch C., Roos E., Pagenstert G., Mündermann A. (2017). Measuring joint kinematics of treadmill walking and running: Comparison between an inertial sensor based system and a camera-based system. J. Biomech..

[46] Zhang J.T., Novak A.C., Brouwer B., Li Q. (2013). Concurrent validation of Xsens MVN measurement of lower limb joint angular kinematics. Physiol. Meas..

[47] Ferrari A., Cutti A.G., Garofalo P., Raggi M., Heijboer M., Cappello A., Davalli A. (2010). First in vivo assessment of outwalk: A novel protocol for clinical gait analysis based on inertial and magnetic sensors. Med. Biol. Eng. Comput..

[48] Cloete T., Scheffer C. Benchmarking of a full-body inertial motion capture system for clinical gait analysis. Proceedings of the 30th Annual International Conference of the IEEE Engineering in Medicine and Biology Society.

[49] Picerno P., Cereatti A., Cappozzo A. (2008). Joint kinematics estimate using wearable inertial and magnetic sensing modules. Gait Post..

[50] Robert-Lachaine X., Mecheri H., Larue C., Plamondon A. (2016). Validation of inertial measurement units with an optoelectronic system for whole-body motion analysis. Med. Biol. Eng. Comput..

[51] Kim S., Nussbaum M.A. (2013). Performance evaluation of a wearable inertial motion capture system for capturing physical exposures during manual material handling tasks. Ergonomics.

[52] Mustonen A.-M., Käkelä R., Joukainen A., Lehenkari P., Jaroma A., Kääriäinen T., Kröger H., Paakkonen T., Sihvo S.P., Nieminen P. (2021). Synovial Fluid Fatty Acid Profiles Are Differently Altered by Inflammatory Joint Pathologies in the Shoulder and Knee Joints. Biology.

[53] Samotus O., Lee J., Jog M. (2021). Developing a Consistent, Reproducible Botulinum Toxin Type A Dosing Method for Upper Limb Tremor by Kinematic Analysis. Toxins.

[54] Pérez-de la Cruz S. (2021). Use of a Portable Inertial Measurement Unit as an Evaluation Method for Supraspinatus Muscle: Proposed Normative Values. Sensors.

[55] Limacher M., Pina I.L., Southard D., Williams M.A., Bazzarre T. (1999). Guidelines for Cardiac Rehabilitation and Secondary Prevention Programs.

[56] Westcott W.L. (2012). Resistance training is medicine: Effects of strength training on health. Curr. Sports Med. Rep..

[57] Chèze L. (2014). Kinematic Analysis of Human Movement.

